# Functional immunity against SARS-CoV-2 in the general population after a booster campaign and the Delta and Omicron waves, Switzerland, March 2022

**DOI:** 10.2807/1560-7917.ES.2022.27.31.2200561

**Published:** 2022-08-04

**Authors:** Rebecca Amati, Anja Frei, Marco Kaufmann, Serena Sabatini, Céline Pellaton, Jan Fehr, Emiliano Albanese, Milo A Puhan, on behalf of the Corona Immunitas Research Group

**Affiliations:** 1 Università della Svizzera Italiana, Facoltà di scienze biomediche, Lugano, Ticino, Switzerland; 2 Epidemiology, Biostatistics & Prevention Institute, University of Zurich, Zurich, Switzerland; 3 Service of Immunology and Allergy, Department of Medicine, Lausanne University Hospital, and University of Lausanne, Lausanne, Switzerland; 4 Members of the Corona Immunitas Research Group are listed under Acknowledgements

**Keywords:** SARS-CoV-2, seroprevalence, neutralising activity, variants of concern

## Abstract

Functional immunity (defined here as serum neutralising capacity) critically contributes to conferring protection against SARS-CoV-2 infection and severe COVID-19. This cross-sectional analysis of a prospective, population-based cohort study included 1,894 randomly-selected 16 to 99-year-old participants from two Swiss cantons in March 2022. Of these, 97.6% (95% CI: 96.8–98.2%) had anti-spike IgG antibodies, and neutralising capacity was respectively observed for 94%, 92% and 88% against wild-type SARS-CoV-2, Delta and Omicron variants. Studying functional immunity to inform and monitor vaccination campaigns is crucial.

Currently available injectable vaccines confer protective immunity against symptomatic severe acute respiratory syndrome coronavirus (SARS-CoV-2) infection and severe coronavirus disease (COVID-19), however, protection – particularly against infection – wanes over time and is reduced for highly mutated variants such as Omicron (Phylogenetic Assignment of Named Global Outbreak (Pango) lineage: B.1.1.529) [1-4]. Booster vaccinations are important to maintain individual protection against severe disease more than infection [5]. Functional immunity (defined here as neutralising capacity of serum) contributes to protection against infection and severe disease, however, neutralising antibodies in serum wane over time, and are less effective at preventing infection by emerging variants. To the best of our knowledge, evidence is inexistent on functional immunity (defined here as neutralising capacity of serum) in the general population after the Delta (Pango lineage: B.1.617.2) and Omicron waves, and after vaccination and booster campaigns. Immunosurveillance of functional immunity is key to plan vaccination campaigns with respect to both their optimal timing and subgroups to be targeted, and to contemplate other preventive measures to control the burden of disease. Our aims were: (i) to determine the proportion of individuals in the general population with functional immunity against SARS-CoV-2, and (ii) to assess the neutralising activity of antibodies for virus variants of concern.

## Study design, sampling, and participants’ characteristics

We conducted a cross-sectional analysis of the baseline assessment of a prospective, population-based cohort study, which is part of the *Corona Immunitas* research programme in Switzerland [6], within which we had completed four phases of seroprevalence studies throughout Switzerland between April 2020 and October 2021 using a standardised protocol. Here we present results from phase five (Figure), for which baseline assessments were done between 1 and 31 March 2022.

**Figure:**
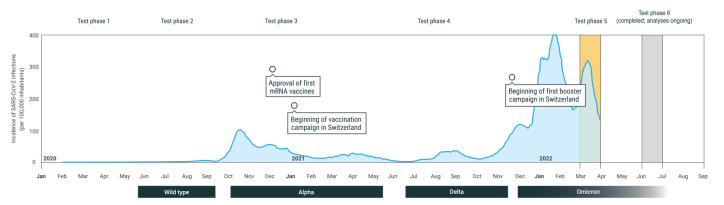
Evolution of the pandemic, timing of vaccination and booster campaign and test phases of *Corona Immunitas*, Switzerland, January 2020–April 2022

The interval from 1 to 31 March 2022 is coloured in orange to highlight the epidemiological context where phase 5 of the *Corona Immunitas* research programme took place. The current study is conducted within this phase 5.

For phase five, we randomly selected individuals from the general population in north-eastern (canton of Zurich) and southern Switzerland (canton of Ticino), two regions that differ across demographic, socio-cultural, linguistic aspects and climate, all of which may impact on the dynamics of the pandemic. The Swiss Federal Office of Statistics provided random samples of the general population in age-stratified (16–29, 30–44, 45–64 and ≥ 65 years) groups, separately for Zurich and for Ticino. We selected these groups after consultation with the Swiss Federal Office of Public Health to adequately account for the potential impact on seroprevalence of social behaviour, adherence to public health measures and vaccination uptake, all of which differ across these age groups. Given a sensitivity of 98% and specificity of 99%, we deemed 200 participants for each stratum to provide precise enough estimates for an expected seroprevalence of 90% or more. The target sample size was thus 200 for each age stratum in the two regions (i.e. total planned sample size of 1,600).

Before the in-person study visit, participants provided information regarding socio-demographics, vaccinations, SARS-CoV-2 infections, hospital and intensive care unit (ICU) admissions and symptoms in case of infections and past medical history, using the secure, web-based Research Electronic Data Capture platform for data collection and management [7,8]. We used questionnaires and data collection procedures identical to the previous phases of *Corona Immunitas* [6] to allow comparability, with small adaptations to the situation of the pandemic in early 2022. We used medians and interquartile ranges or absolute and relative numbers for the descriptive analysis.

We enrolled overall 1,894 individuals (1,044 from north-eastern and 850 southern Switzerland). Participation rate was 21.4% (1,044/4,879) in north-eastern and 18.9% (850/4,497) in southern Switzerland. Women, individuals aged between 45 and 64 years, and persons with high education and socioeconomic status and vaccination were slightly over- and persons with previous infections underrepresented (Table 1).

**Table 1:** Characteristics of the sample, stratified by canton and age group, Ticino and Zurich, Switzerland, March 2022 (n = 1,894)

Study site		Ticino					Zurich				
**Age category in years**		All	16–29	30–44	45–64	≥ 65	All	16–29	30–44	45–64	≥ 65
**Sample size**		850	176	208	262	204	1,044	183	260	326	275
**Median age in years**		48	23	38	54	72	50	24	37	55	72
**IQR **		32–64	19–27	34–42	49–58	68–77	34–65	21–27	33–41	51–59	68–77
**Age group in years**											
16–29	Number	176	176	0	0	0	183	183	0	0	0
	Percentage	20.7	100.0	0.0	0.0	0.0	17.5	100.0	0.0	0.0	0.0
30–44	Number	208	0	208	0	0	260	0	260	0	0
	Percentage	24.5	0.0	100.0	0.0	0.0	24.9	0.0	100.0	0.0	0.0
45–64	Number	262	0	0	262	0	326	0	0	326	0
	Percentage	30.8	0.0	0.0	100.0	0.0	31.2	0.0	0.0	100.0	0.0
≥ 65	Number	204	0	0	0	204	275	0	0	0	275
	Percentage	24.0	0.0	0.0	0.0	100.0	26.3	0.0	0.0	0.0	100.0
**Sex^a^**											
Female	Number	484	103	125	152	104	567	106	157	169	135
	Percentage	56.9	58.5	60.1	58.0	51.0	54.3	57.9	60.4	51.8	49.1
**Educational level**											
Primary	Number	89	39	6	10	34	71	31	7	10	23
	Percentage	10.5	22.2	2.9	3.8	16.7	6.8	16.9	2.7	3.1	8.4
Secondary	Number	475	90	82	180	123	428	79	68	127	154
	Percentage	55.9	51.1	39.4	68.7	60.3	41.0	43.2	26.2	39.0	56.0
Tertiary	Number	278	46	120	70	42	537	73	181	187	96
	Percentage	32.7	26.1	57.7	26.7	20.6	51.4	39.9	69.6	57.4	34.9
Missing	Number	8	1	0	2	5	8	0	4	2	2
	Percentage	0.9	0.6	0.0	0.8	2.5	0.8	0.0	1.5	0.6	0.7
**Household income (CHF/month)**											
0–6,000	Number	371	79	64	107	121	353	97	50	62	144
	Percentage	43.6	44.9	30.8	40.8	59.3	33.8	53.0	19.2	19.0	52.4
6,000–12,000	Number	306	55	100	96	55	392	43	108	143	98
	Percentage	36.0	31.2	48.1	36.6	27.0	37.5	23.5	41.5	43.9	35.6
12,000–18,000	Number	59	13	20	24	2	173	30	60	70	13
	Percentage	6.9	7.4	9.6	9.2	1.0	16.6	16.4	23.1	21.5	4.7
≥ 18,000	Number	45	7	15	16	7	75	7	30	36	2
	Percentage	5.3	4.0	7.2	6.1	3.4	7.2	3.8	11.5	11.0	0.7
Missing	Number	69	22	9	19	19	51	6	12	15	18
	Percentage	8.1	12.5	4.3	7.3	9.3	4.9	3.3	4.6	4.6	6.5
**Employment status**											
Working	Number	529	126	183	208	12	731	177	237	292	25
	Percentage	62.2	71.6	88	79.4	5.9	70	96.7	91.2	89.6	9.1
Missing	Number	5	1	1	1	2	8	0	4	1	3
	Percentage	0.6	0.6	0.5	0.4	1.0	0.8	0.0	1.5	0.3	1.1
**Swiss citizenship**											
Swiss citizen	Number	677	161	138	212	166	880	157	180	284	259
	Percentage	79.6	91.5	66.3	80.9	81.4	84.3	85.8	69.2	87.1	94.2
Missing	Number	3	0	0	2	1	6	0	3	1	2
	Percentage	0.4	0.0	0.0	0.8	0.5	0.6	0.0	1.2	0.3	0.7
**Lifestyle and conditions**											
**Smoking**	Number	178	56	47	52	23	192	37	54	70	31
	Percentage	20.9	31.8	22.6	19.8	11.3	18.4	20.2	20.8	21.5	11.3
Missing	Number	3	0	0	1	2	5	0	1	1	3
	Percentage	0.4	0.0	0.0	0.4	1.0	0.5	0.0	0.4	0.3	1.1
**Obese (BMI ≥ 30)**	Number	102	8	24	37	33	132	11	27	55	39
	Percentage	12	4.5	11.5	14.1	16.2	12.6	6	10.4	16.9	14.2
Missing	Number	1	0	0	1	0	2	0	1	1	0
	Percentage	0.1	0.0	0.0	0.4	0.0	0.2	0.0	0.4	0.3	0.0
** ≥ 1 chronic condition**	Number	188	14	16	56	102	283	13	30	81	159
	Percentage	22.1	8	7.7	21.4	50	27.1	7.1	11.5	24.8	57.8
Missing	Number	1	0	0	0	1	2	0	1	1	0
	Percentage	0.1	0.0	0.0	0.0	0.5	0.2	0.0	0.4	0.3	0.0
**Previous SARS-CoV-2 infection**											
**Positive test (ever)**	Number	356	91	112	111	42	339	76	107	100	56
	Percentage	41.9	51.7	53.8	42.4	20.6	32.5	41.5	41.2	30.7	20.4
Missing	Number	0	0	0	0	0	3	0	1	1	1
	Percentage	0.0	0.0	0.0	0.0	0.0	0.3	0.0	0.4	0.3	0.4
**Infected recently (NuC antibody positive)**	Number	230	64	71	61	34	238	52	73	79	34
	Percentage	27.1	36.4	34.1	23.3	16.7	22.8	28.4	28.1	24.2	12.4
**Infected recently (NuC antibody positive or positive test 2022)**	Number	318	86	102	92	38	318	71	99	102	46
	Percentage	37.4	48.9	49	35.1	18.6	30.5	38.8	38.1	31.3	16.7
**Past severe infections 2020–2022^b^**	Number	13	0	0	4	9	6	0	0	1	5
	Percentage	1.5	0.0	0.0	1.5	4.4	0.6	0.0	0.0	0.3	1.8
Missing	Number	1	0	0	1	0	2	0	0	1	1
	Percentage	0.1	0.0	0.0	0.4	0.0	0.2	0.0	0.0	0.3	0.4
**Vaccination against SARS-CoV-2**											
**Vaccinated (≥ 1 dose)**	Number	765	158	184	237	186	972	169	238	298	267
	Percentage	90	89.8	88.5	90.5	91.2	93.1	92.3	91.5	91.4	97.1
Missing	Number	6	1	0	3	2	4	1	2	1	0
	Percentage	0.7	0.6	0.0	1.1	1.0	0.4	0.5	0.8	0.3	0.0
**Booster dose**	Number	494	74	114	164	142	758	122	178	240	218
	Percentage	58.1	42	54.8	62.6	69.6	72.6	66.7	68.5	73.6	79.3
Missing	Number	100	19	24	27	30	79	14	23	30	12
	Percentage	11.8	10.8	11.5	10.3	14.7	7.6	7.7	8.8	9.2	4.4

BMI: body mass index; CHF: Swiss francs; ICU: intensive care unit; IQR: interquartile range; NuC: nucleocapsid; SARS-CoV-2: severe acute respiratory coronavirus 2.

**^a^** Sex was collected as a binary variable (male; female).

^b^ Severe infections were defined as those requiring a hospital admission (among those three ICU admissions in participants from Ticino and none in Zurich).

## Ascertainment and prevalence of SARS-CoV-2 antibodies

Participants came to onsite visits at a healthcare facility or were offered at-home visits. For each participant, trained personnel collected venous blood samples, according to clinical standards and COVID-19 hygiene measures. We assessed SARS-CoV-2 specific antibodies against the spike and nucleocapsid proteins using Sensitive Anti-SARS-CoV-2 Spike Trimer Immunoglobulin Serological (SenASTrIS), a Luminex binding assay [9]. The assay measures binding of IgG antibodies to the trimeric SARS-CoV-2 spike and the nucleocapsid proteins. The test has a high specificity (98%) and sensitivity (99%) and has been validated in samples of the general population and in specific subgroups [9].

We calculated seroprevalence using a Bayesian logistic regression model accounting for the psychometric characteristics of the serological test and applied post-stratification weights based on the target population demographic structure [10]. We conducted all analyses in R, version 4.1.2.

We found that 97.6% (95% credible interval (CI): 96.8–98.2%) had developed IgG antibodies against the spike protein following vaccination and/or infection (Table 2) without relevant differences across age groups and region. Overall, 34% (636/1,894) of the sample originated from people recently infected, based on a self-reported positive laboratory viral test since January 2022, and/or detection of anti-nucleocapsid IgG antibodies.

**Table 2:** Prevalence of SARS-CoV-2 IgG antibodies and ACE2r-blocking (neutralising capacity) as measured by a virus-free assay, stratified by canton and age group, Ticino and Zurich, Switzerland, March 2022 (n = 1,894)

Study site		Ticino					Zurich				
**Age groups in years** **(total number of individuals)**		All (n = 850)	16–29 (n = 176)	30–44 (n = 208)	45–64 (n = 262)	≥ 65 (n = 204)	All (n = 1,044)	16–29 (n = 183)	30–44 (n = 260)	45–64 (n = 326)	≥ 65 (n = 275)
**Presence of anti-spike IgG antibodies**											
Number		822	172	202	253	195	1027	179	257	321	270
%		96.7	97.7	97.1	96.6	95.6	98.4	97.8	98.8	98.5	98.2
**Level of anti-spike IgG antibodies^a^**											
Not detectable	Number	28	4	6	9	9	17	4	3	5	5
	%	3.3	2.3	2.9	3.4	4.4	1.6	2.2	1.2	1.5	1.8
Low (≥ 6 − < 12)^b^	Number	11	6	2	3	0	12	3	4	4	1
	%	1.3	3.4	1.0	1.1	0.0	1.1	1.6	1.5	1.2	0.4
Moderate (≥ 12 − < 40)^b^	Number	28	6	10	9	3	20	3	6	7	4
	%	3.3	3.4	4.8	3.4	1.5	1.9	1.6	2.3	2.1	1.5
High (≥ 40)^b^	Number	783	160	190	241	192	995	173	247	310	265
	%	92.1	90.9	91.3	92.0	94.1	95.3	94.5	95.0	95.1	96.4
U/mL according to Elecsys anti-SARS-CoV-2 S^c^	Median	2,511	2,443	2,624	2,534	2,453	2,637	2,637	2,707	2,605	2,637
	IQR	2,139–3,062	2,054–3,131	2,142–3,175	2,140–2,913	2,194–2,953	2,143–3,051	2,202–3,127	2,151–3,069	2,070–3,012	2,139–3,029
**Seroprevalence**											
%		97.5	97.1	98.3	97.9	97.2	98.8	98.1	99.3	99.1	98.8
95% CrI in %		95.8–99	94.3–98.8	95.6–99.7	95.2–99.6	93.9–99.3	97.9–99.5	96–99.3	97.8–99.9	97.6–99.8	96.8–99.7
**Neutralisation**											
Wild type	Number	787	161	190	243	193	996	173	247	312	264
	%	92.6	91.5	91.3	92.7	94.6	95.4	94.5	95.0	95.7	96.0
Delta	Number	774	159	188	239	188	978	172	246	305	255
	%	91.1	90.3	90.4	91.2	92.2	93.7	94.0	94.6	93.6	92.7
Omicron	Number	742	154	183	228	177	934	167	233	290	244
	%	87.3	87.5	88.0	87.0	86.8	89.5	91.3	89.6	89.0	88.7

ACE2r: angiotensin-converting enzyme 2 receptor; CrI: credible interval; IgG: immunglobulin G; IQR: interquartile range; MFI: mean fluorescence intensities; NuC: nucleocapsid.

^a^ Unit for levels of anti-spike IgG antibodies is the MFI as measured by the Luminex binding assay Sensitive Anti-SARS-CoV-2 Spike Trimer Immunoglobulin Serological (SenASTrIS) [9].

^b^ Low: from threshold of test positivity (MFI ≥ 6) to < 3 standard deviations above this threshold (MFI < 12); moderate: ≥ 3 standard deviations above positivity (MFI ≥ 12) threshold but unlikely to provide neutralisation (MFI < 40); high: neutralising capacity likely (MFI ≥ 40).

^c^ For interpretation of quantitative results MFI values were converted to U/mL as measured by the Elecsys Anti-SARS-CoV-2 immunoassay produced by Roche. Roche anti-S IgG = 10^(−0.6108069 + 2.0072882 × log10(MFI  + 1)) as developed by the Department of Clinical Immunology & Allergy of the University Hospital of Lausanne based on population-based samples.

## Ascertainment and prevalence of neutralising capacity against wild-type SARS-CoV-2 as well as Delta and Omicron variants

We also assessed the presence of SARS-CoV-2 neutralising antibodies using a cell- and virus-free assay [11]. This assay measures the proportion of antibodies that block the interaction of the angiotensin-converting enzyme 2 receptor (ACE2r) with the receptor-binding domain of the trimer spike protein of the wild type and variants of concern.

The proportion of individuals whose antibodies showed ACE2r-blocking capacity in this virus-free assay was high against the wild-type SARS-CoV-2 (1,783/1,894; 94%), and Delta variant (1,752/1,894; 93%) and appeared only slightly lower for the Omicron (1,676/1,894; 88%), with no relevant differences across the age groups, but slightly higher proportions in north-eastern compared with southern Switzerland (Table 2). When stratified for recent infection, we found that more participants with anti-nucleocapsid IgG antibodies seemed to show ACE2r-blocking capacity against Omicron than those without (96% (221/230) vs 84% (521/620) in Ticino and 93% (222/238) vs 88% (712/806) in Zurich). In contrast, for wild-type SARS-CoV-2 and Delta variant, ACE2r-blocking capacities against Omicron remained similar, whether anti-nucleocapsid IgG antibodies were present or not. The proportions of participants with anti-nucleocapsid IgG antibodies also appeared to decrease across groups with increasing age (Table 1).

## Discussion

The introduction of vaccines against SARS-CoV-2 and the circulation of highly infectious but less virulent variants of concern, including Omicron, have considerably contributed to reducing the burden of COVID-19 on individuals and health services. Infection spreading is still substantial, but hospital and ICU admissions, and mortality rates have steadily decreased in many countries, including in Switzerland, since late December 2021 [12,13]. It is plausible that seroprevalence (i.e. the proportion of individuals with anti-spike SARS-CoV-2 antibodies) exceeds 90% for the adult population in countries that were considerably exposed to natural infection and attained high vaccination coverage at the same time [14].

Indeed, by March 2022, almost the entire population in the current study developed antibodies against SARS-CoV-2, irrespective of age and region of residence in Switzerland. The vast majority of individuals also developed antibodies with neutralising capacity against the wild type virus, as well as the Delta and Omicron variants. Neutralising antibodies are critical for protection against infection and play an important role in protection against severe disease [15,16]. Our findings additionally suggest that a substantial part of the general population in Switzerland developed functional hybrid immunity as a result of infection and vaccination. Of note and as suggested by an apparent differential proportion of anti-nucleocapsid IgG antibodies across age groups (Table 1), a smaller proportion of the elderly population might have had hybrid functional immunity potentially widening the immunity gap over time.

To the best of our knowledge, no population-based seroprevalence studies conducted in European countries in 2022 have been published to date. However, our findings on seroprevalence nearing 100% are expected, as in line with the increase already reported in European [14] and non-European countries [17,18]. A remarkable result of this study is the high proportion of the population whose antibodies showed neutralising activity against different variants of SARS-CoV-2, including Omicron. This may be due to the booster campaign offered in late autumn of 2021, but it is also likely ascribable to the high incidence of infections caused by both Delta and Omicron variants in late 2021 and early 2022 (Figure), when more stringent public health measures were progressively relaxed. The combination of infections and booster vaccinations likely explains the high prevalence of functional immunity at present.

This study has three major strengths: (i) we did a cross-sectional analysis of a prospective, population-based study, whereas population-based studies on SARS-CoV-2 neutralising antibodies are almost inexistent in Europe [19]; (ii) we adopted a standardised protocol and antibody test, across sites and time, since the beginning of the pandemic; (iii) the current study was timely in March 2022, which was a few weeks after very high incidence of infections due to the Delta and Omicron BA.1 and BA.2 subvariants (Figure). Limitations include the low participation rate and overrepresentation of people with higher education and socioeconomic status. However, while vaccination uptake may be higher, infection rates may have been lower in this group compared with the entire population. Other limitations include the limited scope of immune function assessed (e.g. no T-cell function) and the possibility that (future) variants may evade neutralisation as assessed here. Future immuno-epidemiological studies may also assess mucosal IgA and tissue resident cellular immunity, which are not induced by current injectable vaccines, but increasingly recognised as important for consideration in future vaccines [20].

## Conclusion

In conclusion, antibody response and neutralising capacity are both very high in the Swiss population after the booster campaign in late 2021, and after high rates of infections due to the Delta and Omicron variants of SARS-CoV-2. This results in robust protective immunity. The temporal trajectory of protective immunity must be monitored to determine if, when and to whom booster vaccinations should be offered.
